# Impacts of sampling effort on seasonal plant-pollinator interaction turnover over eight years

**DOI:** 10.1007/s00442-025-05771-8

**Published:** 2025-07-11

**Authors:** Isabella Manning, Leana Zoller, Julian Resasco

**Affiliations:** Department of Ecology and Evolutionary Biology, University of Colorado, Boulder, CO 80309 USA

**Keywords:** Community ecology, Interaction rewiring, Networks, Beta-diversity, Temporal dynamics

## Abstract

Plant-pollinator interaction networks are dynamic in time and space. Interaction turnover consists of interaction rewiring (i.e., changes in interactions independent of species turnover) and species turnover (i.e., the gain or loss of species present in the network). To capture network dynamics, it is crucial to address the effect of sampling effort because insufficient data can distort apparent network patterns. We used eight years of plant-pollinator interaction data from a subalpine meadow to examine patterns of temporal (week-to-week) interaction turnover and the role of sampling effort. With increasing sampling effort, values of interaction turnover and species turnover decreased, and rewiring increased. Saturation curves suggest an approach towards true values with higher sampling effort. Across the eight years, substantial variation in weekly and seasonal interaction turnover was observed, with identifiable seasonal trends across all aggregated years. These results demonstrated that the interpretation of interaction turnover and its components is sensitive to sampling effort, stressing the importance of considering its role in network studies.

**Supplementary Information:**

The online version contains supplementary material available at 10.1007/s00442-025-05771-8.

## Introduction

Plants and pollinators engage in mutualistic interactions that are beneficial to the plant and the pollinating animal (Bascompte 2009). These plant-pollinator interactions form and dissolve over time and space, creating a dynamic network structure (CaraDonna et al. 2021). In temperate areas, where cold weather limits the growth of plants and pollinators, flowers and pollinator activity generally increase as the weather warms throughout the season, then decrease as the season wanes and temperatures drop (Inouye 2022). Across a growing season and among years, the phenologies of plants and their pollinators are variable–at different times flowers bloom, insects emerge, become active, and die. These phenological processes influence opportunities for interactions as species enter or leave the network over the season (Simanonok and Burkle 2014; Ogilvie and Forrest 2017; Resasco et al. 2021). Pollinator preferences for floral resources may also vary throughout the season (Ogilvie and Thomson 2016). There has been increasing interest in understanding fluctuations within these networks and the underlying processes driving them (Poisot et al. 2015; Trøjelsgaard and Olesen 2016; CaraDonna et al. 2017; Chacoff et al. 2018; Schwarz et al. 2020; Resasco et al. 2021).

**Fig. 1:**
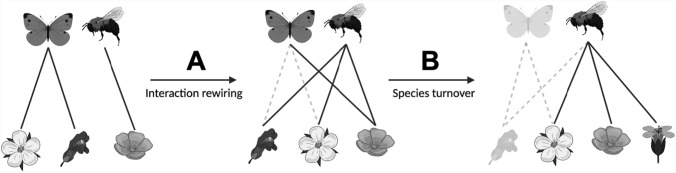
Conceptual diagram of interaction rewiring and species turnover. Solid lines indicate conserved or new interactions, while dotted lines represent lost interactions. **A** shows interaction rewiring as changes only in which species are interacting over time. **B** shows species turnover as changes in the species present or gain or loss of species over time and the associated changes in interactions. Diagram adapted from CaraDonna et al. 2017

Interactions may change over time and space due to two observable patterns: (1) interaction rewiring, as the change in interactions independent of species turnover, or (2) species turnover, as the change in species present (Poisot et al. 2012). Interaction rewiring represents the reassembly of interactions among shared species over time and space, while species turnover represents changes in community composition over time and space as species are gained or lost (Fig. 1). Together, these measures provide insight into the dynamics of plant-pollinator interactions and allow examination of the mechanisms driving changes in community structure and function. The patterns of interaction dissimilarity or turnover in plant-pollinator networks can be examined by utilizing beta-diversity, which quantifies changes in species diversity (Novotny 2009; Poisot et al. 2012). Beta-diversity, broadly defined as the turnover of ecological entities (e.g., species or interactions) between sampling units (Novotny 2009; Anderson et al. 2011), can also be used to assess dissimilarity in interaction networks (Poisot et al. 2012; Simanonok and Burkle 2014; Carstensen et al. 2014; CaraDonna et al. 2017). In this study, interaction turnover assesses the dissimilarity between pairs of networks sampled one week apart from each other across growing seasons.

An important consideration in the investigation of network dynamics is the impact of sampling effort (Schwarz et al. 2020). Incomplete sampling may affect inferences from collected data and bias interpretation of network measures (Vázquez et al. 2022; Blüthgen and Staab 2024), including interaction turnover and its components. While fully sampling plant-pollinator networks in nature is practically impossible in most systems, researchers may increase sampling effort to obtain a more complete picture of the network (Jordano 2016). By understanding how sampling effort affects inferences about network measures and their dynamics, researchers can better understand potential biases and guide their study design (Jordano 2016). Although the effect of sampling effort has been considered on some network metrics (Nielsen and Bascompte 2007; Chacoff et al. 2012; Rivera-Hutinel et al. 2012; Vizentin‐Bugoni et al. 2016), its influence on the dynamics of interaction turnover is poorly understood.

In this study, we used eight years of plant-pollinator interaction data to examine how sampling effort (as the number of plots included in the analysis) affects the interpretation of and assumptions about the components of interaction week-to-week turnover and the patterns observed across seasons and years. We asked (1) how apparent interaction turnover and its components (i.e., species turnover and interaction rewiring) change as a function of sampling effort, and (2) how interaction turnover and its components change across seasons and over eight years. To investigate these questions, we used two different methods to calculate the components of interaction turnover, one proposed by Poisot et al. (2012) and the other proposed by Fründ (2021); different methods of calculating interaction turnover may also affect interpretations of the magnitude and variability of interaction turnover components (Poisot et al. 2012; Fründ 2021).

With these questions in mind, we hypothesized that (1) sampling effort influences the interpretation of interaction turnover and its components. As sampling effort increases, more species and interactions are detected, reducing apparent interaction turnover. Since species richness saturates faster than interaction richness with greater sampling effort (Resasco et al. 2021), the contribution of species turnover declines with increasing sampling effort. Given the additive relationship between species turnover and rewiring (Poisot et al. 2012), a reduction in species turnover results in a proportional increase in rewiring. We also hypothesized that (2) interaction turnover is expected to follow a relatively consistent seasonal pattern across years, decreasing towards the end of the season as species and interactions become more persistent. Rewiring may follow an inverse U-shaped trend, peaking in the middle of the flowering season when species turnover is lowest. In contrast, species turnover may be higher at the beginning and end of the season, reflecting rapid changes in floral and pollinator composition driven by phenology and environmental factors such as snowmelt and temperature. However, these patterns may vary across years due to variability in climate, resource availability, and idiosyncrasies of small networks.

## Methods

### Sampling and collection

The study was conducted in a subalpine meadow (Fig. S1) in the Colorado Rocky Mountains, at the University of Colorado’s Mountain Research Station (40°01048′′ N, 105°32026′′ W). Data were collected weekly (at an average of 7.06 days apart) during the annual flowering season from 2015 through 2022; the flowering season typically begins after snowmelt in late May or early June, with plant, pollinator, and interaction richness peaking in mid-summer, and the season concluding in September (Fig. S2). Sampling was conducted in six plots (five 30 × 2 m plots and one 20 × 2 m plot) across the meadow, which occupies an area of approximately 110 × 40 m (Fig. S1). Interactions were recorded during fair weather between 8:00 and 12:00 h. Plant-pollinator interactions were sampled by walking around each plot for 15 min in random order and observing flowers for visitors. All plots were sampled each week for exactly the same duration (15 min) such that the sampling effort was consistent across the season. When a pollinator contacted the reproductive part of the flower, the pollinator was collected with an aspirator or net. The pollinator was identified when it was collected or later identified in the lab, with the plant species recorded at the time of sampling. A total of 81% of pollinators were identified to species level, and 18% were identified to genus level. Taxonomic resolution of identifications can also play a role in network inference (Vázquez et al. 2022); for example, species identified to the genus level may mask species turnover and influence turnover components. This study builds on the dataset from Resasco et al. (2021), including three additional years of sampling.

### Partitioning interaction turnover

Interaction turnover (ßWN, using Whittaker's dissimilarity), a measure of beta diversity, or the dissimilarity between networks, can be partitioned through differing calculation methods (Poisot et al. 2012; Fründ 2021), but each has the practical objective of separating its components of interaction rewiring and species turnover. Specifically, these components are (1) the rewiring component (ßOS), which is the dissimilarity of interactions between species shared by both networks, and (2) the species turnover component (ßST), which is the dissimilarity of interactions due to species turnover (Poisot et al. 2012). To separate these components, Poisot et al. (2012) proposed a method that derives its partitioning via an additive model in which interaction rewiring (ßOS) is subtracted from the overall interaction turnover (ßWN) to obtain species turnover (ßST) so that ßWN = ßST + ßOS. An alternative method derived from an additive partitioning approach based on Novotny (2009) has also been suggested (Fründ 2021). This method keeps the same denominator for the total network dissimilarity, only partitioning the numerator, which results in lower magnitudes of interaction rewiring. Fründ (2021) intends to partition dissimilarity into additive components and contends that the method proposed by Poisot et al. (2012) is not properly additive and overestimates the role of rewiring. Hereafter, the method proposed by Poisot et al. (2012) will be referred to as the “poisot” method while the method proposed by Fründ (2021) will be referred to as the “commondenom” method, each referring to how they are identified in the R package “bipartite” (v 2.18, Dormann et al. 2009). Because the methods of calculating ßOS and ßST may address different aspects of questions about plant-pollinator network dissimilarity (Fründ 2021), it is worthwhile considering both partitioning methods to examine whether the magnitude or variation of values of ßOS and ßST change for each of the research questions. This study used empirical data to compare these methods to better understand how week-to-week interaction turnover changes as a function of sampling effort and assess whether the conclusions are qualitatively similar.

### Statistical analyses

To calculate interaction turnover (ßWN) and its two components of interaction rewiring (ßOS) and species turnover (ßST), weekly plant-pollinator interaction matrices were created for each of the eight years to measure turnover across week-to-week interaction networks. The number of weeks sampled in each year ranged from between 13 and 17. Only weekly networks were included in the analyses that consisted of at least three plant species and three pollinator species, resulting in a total of 117 weekly networks. In plant-pollinator interaction matrices, columns represent pollinator species, rows represent plant species, and values represent the number of observed interactions. Then, ßWN, ßOS, and ßST values were calculated for all subsequent weekly matrices using the *betalinkr* function implemented in the R package “bipartite” (v 2.18, Dormann et al. 2009). ßWN, ßOS, and ßST were calculated using both the “poisot” (Poisot et al. 2012) and “commondenom” (Fründ 2021) partitioning methods. All analyses were performed using values obtained by the two methods. All statistical analyses were performed in R version 4.3.0 (R Core Team 2023).

Sampling effort in interaction network studies is often increased by expanding the area (Dáttilo et al. 2019) or duration of sampling (e.g., Vizentin-Bugoni et al. 2016). To examine the effect of increased sampling effort, calculation of ßWN, ßOS, and ßST was repeated on subsets of data, including different numbers of plots, with increasing plots increasing both area and duration of sampling. To calculate these values, subsets of data were created by including every combination of one to six plots (e.g., for one: each plot individually, for two: plots 1 and 2, plots 1 and 3, plots 2 and 3… and so on), until values for all 126 unique combinations of plots had been obtained.

### The effect of sampling effort on apparent interaction turnover and its components

The first aim was to examine how apparent interaction turnover and its components of interaction rewiring and species turnover change as a function of sampling effort. Specifically, we were interested in determining how average values of ßWN, ßOS, and ßST changed across the season, aggregated across years, depending on the number of plots sampled. To investigate this question, a series of multiple linear regression models were fit using the *lm* function in the “stats” R package (version 4.3.0, R Core Team 2023). Average values of ßWN, ßOS, and ßST, respectively, were included as response variables, and ordinal day (i.e., calendar day of year ranging from 1 to 366 starting on January 1) and the number of included plots as predictors. Initially, the interaction between the two predictor variables was also included to examine whether the effects of sampling effort vary over the season. To check for multicollinearity in the models, we calculated the generalized Variance Inflation Factor (gVIF). Specifically, we calculated gVIF^(1/(2*Df)), as this provides a more interpretable VIF equivalent. For all three models (using average values of ßWN, ßOS, and ßST as response variables), the VIF equivalent for the interaction term and the number of plots showed a VIF greater than 5, indicating high collinearity. Therefore, we removed the interaction term. After removal of the interaction term, the VIF equivalent of all variables was close to 1, indicating no multicollinearity. The predictor variables were fitted as both linear and quadratic terms, as a non-linear relationship between the response and predictors was predicted. The model fit including the quadratic terms was compared to models where predictors were included only as linear terms using AIC (Akaike information criterion) values. In all cases, models that included a quadratic term were a better fit. Models were visually inspected to ensure that model assumptions were fulfilled, including linearity, homogeneity of variance, collinearity, and normality of residuals, using the *check_model* function in the “performance” R package (version 0.10.8, Lüdecke et al. 2021). We used these models to assess the relationship between sampling effort and turnover metrics, rather than for strict statistical inference, since resampling of plots generates non-independence.

For each final selected model, a comparison of the response variable predicted by each combination of the number of plots included was conducted to further examine the effects of sampling effort. The “emmeans” R package (version 1.8.9, Lenth et al. 2023) was used for comparisons between plot combinations. The “emmeans” package uses the Tukey method for comparing pairs and adjusting p values.

To gain additional insight into how resulting data were affected by sampling effort, species richness and interaction richness were calculated and visualized to see how they increase with increasing sampling effort. Accumulation and extrapolation curves were plotted, using the *iNEXT* function in the iNEXT R package (version 3.0.0, Hsieh et al. 2016).

### Dynamics of interaction turnover and its components across seasons

Our second aim was to examine how interaction turnover and its components change across seasons and over eight years. Specifically, we were interested in determining how flowering season (measured by ordinal day) and year related to values of ßWN, ßOS, and ßST.

To investigate this question, a series of linear mixed-effect models (LMMs) were fit using the *lmer* function in the package “lme4” (version 1.1–35, Bates et al. 2015). ßWN, ßOS, and ßST, respectively, were the response variables, ordinal day was included as a fixed predictor, and year (as a factor with eight levels) was included as a random effect. The predictor variable, ordinal day, was included both as a linear and as a quadratic term, as the relationships were hypothesized to be non-linear. Each model was visually validated to ensure that model assumptions were fulfilled.

## Results

### The effect of sampling effort on apparent interaction turnover and its components

**Fig. 2:**
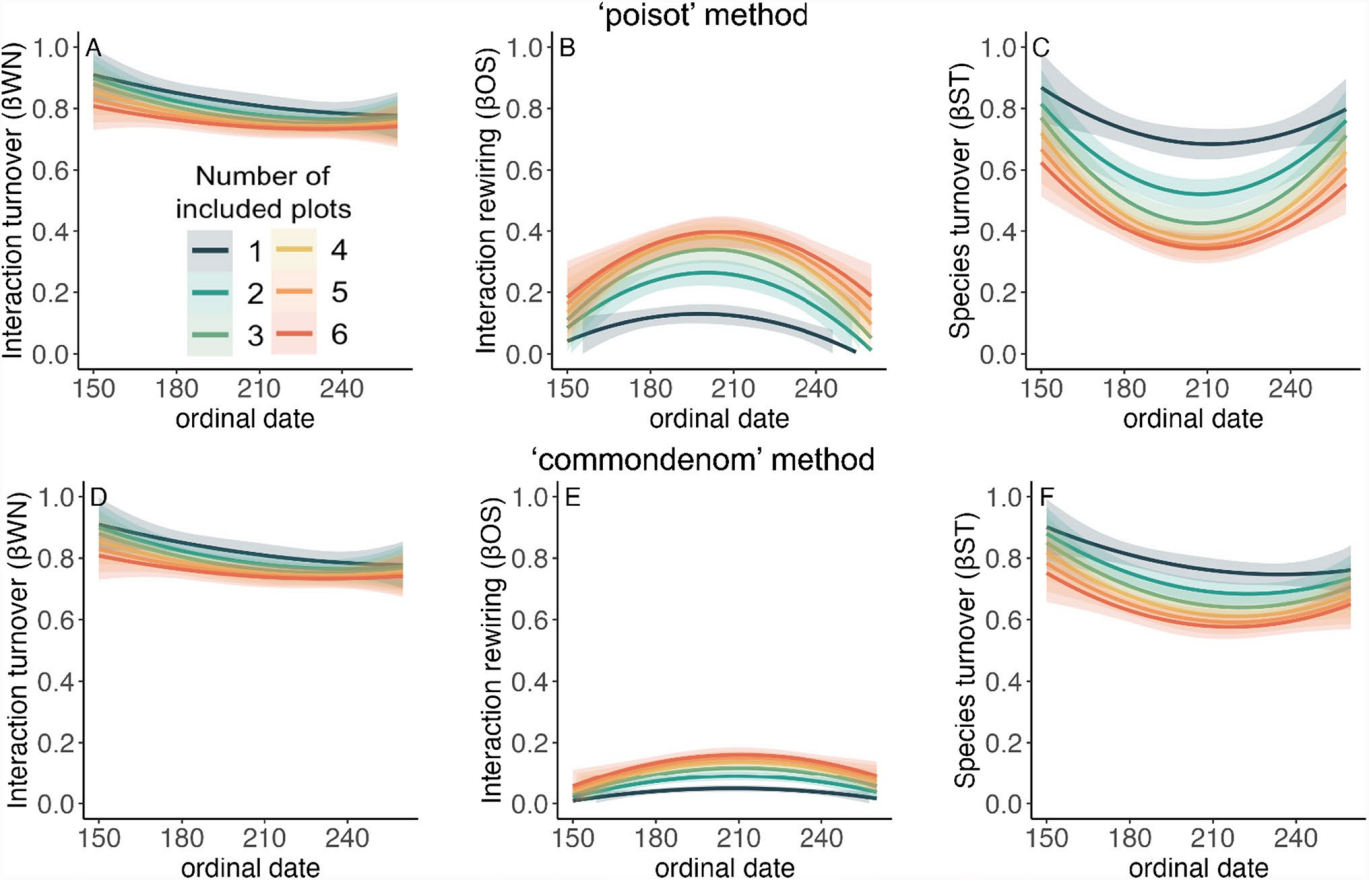
Interaction turnover and its components as a function of sampling effort across the season. The top row (**A**, **B**, and **C**) uses the “poisot” method for partitioning while the bottom row (**D**, **E**, and **F**) uses the “commondenom” method for partitioning. Plots show the relationship between ordinal date and average values of ßWN (**A** and **D**, note that ßWN calculation is identical across methods), ßOS (**B** and **E**), and ßST (**C** and **F**) with polynomial regression lines for different numbers of included plots, from 1 to 6. Shaded areas represent standard error

**Fig. 3:**
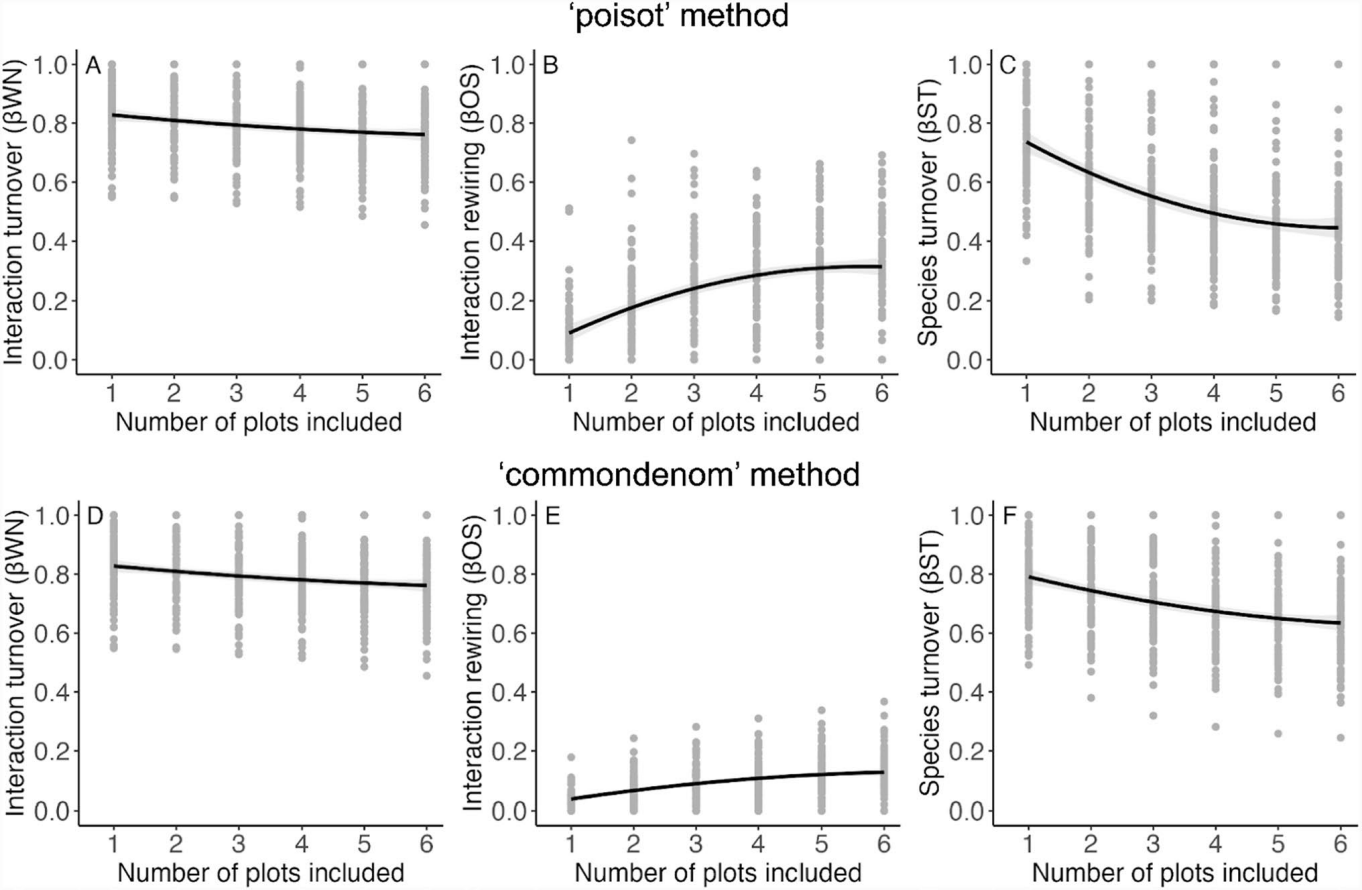
Interaction turnover and its components as a function of sampling effort. The top row (**A**, **B**, and **C**) uses the “poisot” method while the bottom row (**D**, **E**, and **F**) uses the “commondenom” method for turnover partitioning. Plots show the relationship between average values of ßWN (**A** and **D**, note that ßWN calculation is identical across methods), ßOS (**B** and **E**), ßST (**C** and **F**), and the number of plots included in the analysis (sampling effort)

Temporal interaction turnover (i.e., turnover in interactions from one week to the next; ßWN), rewiring (ßOS), and species turnover (ßST) were affected by sampling effort (number of plots included; Figs. 2 and 3). Specifically, interaction turnover and species turnover decreased, and rewiring increased, as the sampling effort increased. A comparison of the response variable predicted by each combination of the number of plots included revealed that as the difference between the number of plots included increased, the difference in the response variable increased (Table S1; Fig. S4).

Apparent interaction turnover decreased as the number of plots included increased (Figs. 2 and 3), and changed across the season depending on the ordinal day (Table S2, Fig. 2). Because the calculation for ßWN is identical in both methods, ßWN exhibits the same trends when the “poisot” method was used as with the “commondenom” method. Apparent interaction rewiring, ßOS, increased as the number of plots included increased (Table S2, Figs. 2 and 3). Finally, apparent species turnover, ßST, decreased as the number of plots included increased (Table S2, Figs. 2 and 3). Results using the “commondenom” method displayed similar trends to the results using the “poisot” method (Table S2, Figs. 2 and 3).

### Dynamics of interaction turnover and its components across seasons

**Fig. 4:**
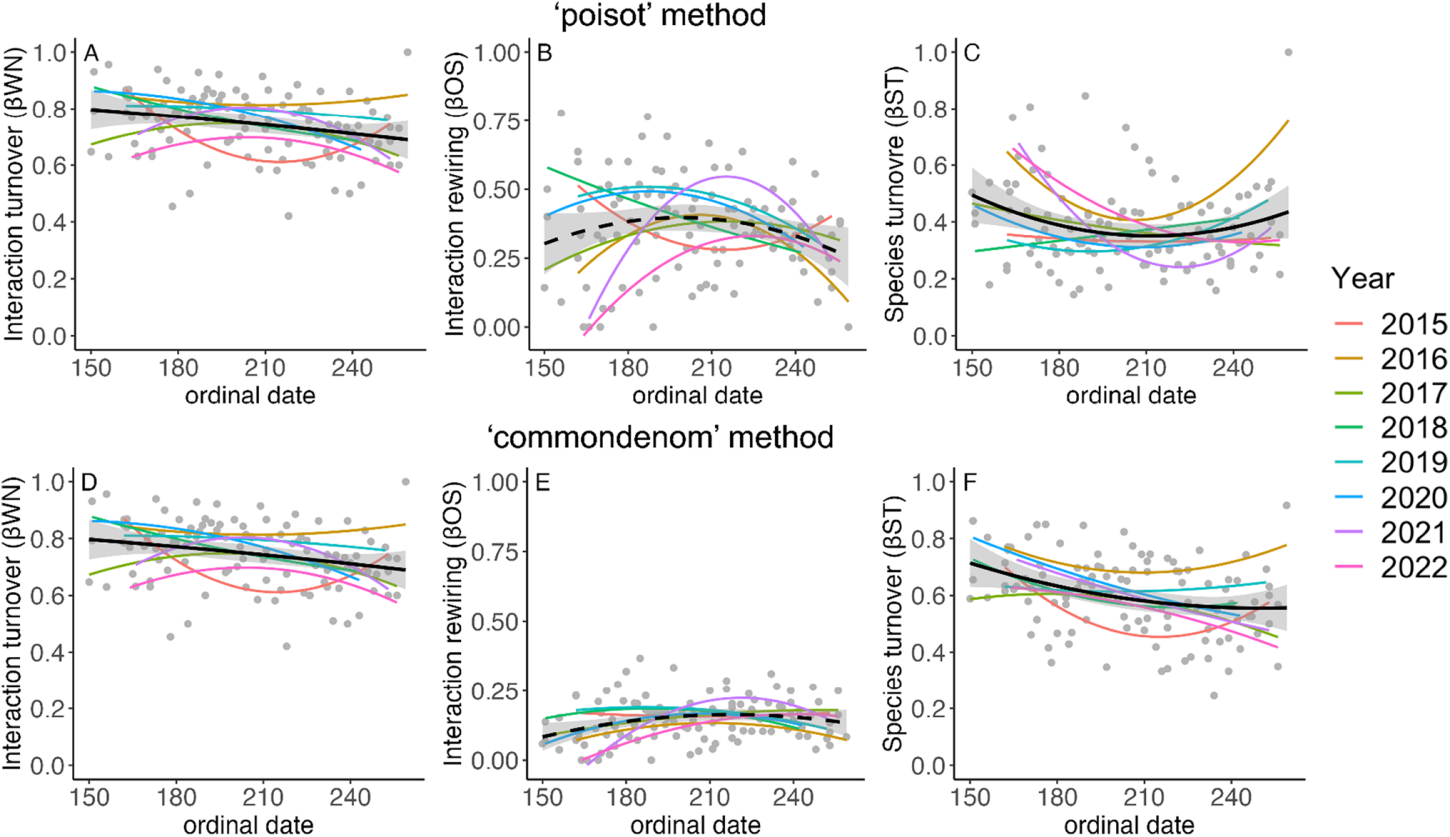
Interannual variation in interaction turnover and its components. The top row (**A**, **B**, and **C**) uses the “poisot” method for partitioning. The bottom row (**D**, **E**, and **F**) uses the “commondenom” method for partitioning. Plots display the relationship between ordinal day and ßWN (**A** and **D**), ßOS (**B** and **E**), and ßST (**C** and **F**), with polynomial regression lines for each year from 2015–2022. Solid black lines indicate a statistically significant relationship; dashed black line indicates a non-significant relationship. Shaded areas represent the confidence interval of the regression line

Interaction turnover, ßWN, varied considerably across years but generally followed a decrease from the beginning to end of a season (Table S3, Fig. 4). Ordinal day as a linear term had a significant negative effect on ßWN (Table S3). Rewiring, ßOS, was highly variable across years but somewhat tended to peak in the middle of the season with a trend toward the lowest values occurring at the beginning and end of the season (Table S3, Fig. 4). However, there was no significant effect of ordinal day (Table S3). Finally, species turnover, ßST, generally had higher values than rewiring at the beginning of the season, declined in the middle of the season, then had a slight uptick towards the end of the season (Table S3, Fig. 4). Ordinal day was positively related to ßST (Table S3). Random effects analysis revealed variance in intercepts across years for all models (Table S3). As the calculation method for ßWN is identical in both methods, ßWN exhibited the same trends when using the “poisot” or “commondenom” methods (Table S3, Fig. 4). When using the “commondenom” method, trends across years were somewhat similar for ßOS and ßST, with ßOS having a lower overall magnitude of contribution to interaction turnover compared to the “poisot” method, and ßST having a higher overall contribution to ßWN (Table S3, Fig. 4).

## Discussion

Our findings revealed that as sampling effort increased, apparent interaction turnover (ßWN) decreased. As sampling effort increased, the apparent levels of the rewiring component (ßOS) also increased, while the apparent levels of the species turnover component (ßST) decreased. However, at a higher level of sampling effort—measured by the number of plots included—the results stabilized, indicating that the network was becoming robustly sampled and approaching saturation. This suggests that, with adequate sampling, it is possible to obtain reliable estimates of interaction turnover and its components (Jordano 2016). The findings also revealed that ßWN, ßOS, and ßST exhibited seasonal patterns; ßWN decreased over the season, ßST was higher towards the beginning and end of the flowering season, and ßOS showed a tendency towards rising in the middle of the season. These patterns are likely driven by seasonal phenology and seasonal variations in climatic and other environmental factors that influence networks. These results were consistent irrespective of the partitioning method (“poisot” or “commondenom”) used.

### The effect of sampling effort on apparent interaction turnover and its components

Because insufficient sampling of plant-pollinator networks can distort approximations of the network patterns (Jordano 2016; Vázquez et al. 2022), understanding how sampling effort affects inferences on network patterns is important. Sampling effort in ecological studies can be varied by increasing either the temporal extent, such as the duration or frequency of surveys, or by increasing the spatial extent, such as the number of plots or transects sampled. In our study, sampling effort is increased by expanding both the spatial and temporal extents of sampling. By incorporating more plots and increasing the sampling extent, researchers can capture greater spatial and temporal heterogeneity, providing more representative ecological networks. Here, the apparent decreases in ßWN and ßST, as well as apparent increases in ßOS with increasing sampling effort, may be explained by species richness becoming saturated more quickly than interaction richness with increased sampling effort (Fig. S3). As species become better sampled with increasing sampling effort, lower species turnover from one week to the next should become more evident. Rare species are more likely to be detected at higher levels of sampling, influencing turnover estimates.

With more complete sampling, more possible pairwise interactions were also revealed. However, because the detection of new interactions did not increase at the same rate as the detection of new species (Fig. S3), the resulting rates of interaction rewiring compared to species turnover were higher when sampling was more complete. Furthermore, pairwise comparisons revealed that as the number of plots included increased, the effect of sampling effort on ßWN, ßOS, or ßST decreased. For instance, comparing five plots to six plots had less of an impact on the metrics than comparing one plot to two. This indicates that sampling effort was approaching a plateau for ßWN, ßOS, or ßST estimates, providing a more reliable depiction of interaction turnover at a higher sampling effort.

The contrasting partitioning methods did not result in any qualitative differences in the overall results. Sampling effort and ordinal day still significantly affected average values of ßWN, ßOS, and ßST, regardless of the calculation method. While ßOS and ßST additively contribute to ßWN in both methods, the partitioning approach led to different magnitudes for each component. For example, when values of ßST were low, values of ßOS were high, an attribute of partitioning that is particularly visible using the “poisot” method. The “commondenom” method reduced apparent rewiring, so while the two components are still additive, they are partitioned differently using a common denominator and ßOS is likely to display an inherently lower magnitude, which was reflected in the results. Pairwise comparisons revealed fewer significant differences in ßOS and ßST with the “commondenom” method compared to the “poisot” method, indicating a slightly less pronounced effect of sampling effort. However, the general observed patterns were consistent between the two methods.

### Dynamics of interaction turnover and its components across seasons

There was evidence of seasonal interaction turnover displaying prominent patterns across eight years, with interaction turnover (ßWN) decreasing from the beginning to the end of the season when yearly variation was controlled for. The relatively higher degree of interaction turnover at the beginning of the season is likely due to species’ phenological differences and high variation in emergence time for different species at the beginning of the season (e.g., early season flowers such as *Cymopterus lemmonii* and *Taraxacum officinale* and early season pollinators like *Osmia bucephala*; Simanonok and Burkle 2014, Resasco et al. 2021). At this point in the season, turnover is high, and new species emerge and enter the network each week. At the approximate midpoint of the season, most plant species are flowering and pollinator species are active, decreasing week-to-week overall dissimilarity. For rewiring (ßOS), a trend of relatively higher rewiring during the peak of the season was detected, which may be due to increased options for interactions between plants and pollinators to reassemble in the middle of the season when species richness of plants and pollinators are at their highest. Species turnover (ßST) was significantly affected by ordinal day and displayed an opposite trend to rewiring across all years, due to the additive nature of the two components.

The finding of aggregated seasonal trends in each interaction turnover component is consistent with the results of CaraDonna et al. (2017) on seasonal plant-pollinator interaction turnover across three years. However, our study demonstrated a greater degree of variability in inter-annual week-to-week temporal dynamics. While this study reflected the general seasonal trends identified by CaraDonna et al. (2017), the same degree of consistency across all eight years was not observed. This may suggest that seasonal plant-pollinator network dynamics may not be as consistent across years as previously documented.

### Partitioning method

We examined the difference in calculation methods of partitioning ßWN into ßOS and ßST. A few recent studies have compared the two methodologies based on Poisot et al. (2012) and Fründ (2021). Ceron et al. (2022) used both methods in their predator–prey network study and found no changes to their main conclusions based on the partitioning method. Lázaro and Gómez‐Martínez (2022) considered both partitioning methods for estimating the rate of rewiring of plant-pollinator networks along a gradient of habitat loss, but lacked discussion of differences between the two methods. Whereas our analyses identified some differences between partitioning methods, overall conclusions regarding the effects of sampling effort and patterns of seasonal dynamics of ßWN and its components did not change substantially. This study contributes to the understanding of how interaction turnover partitioning methodology influences the interpretation of plant-pollinator network patterns.

## Conclusion

This study calls further attention to the role of sampling effort in plant-pollinator network studies and how sensitive interaction turnover and its components are to incomplete sampling. Insufficient sampling effort can lead to overestimation of interaction turnover (ßWN) and the species turnover component (ßST), as well as underestimation of the rewiring component (ßOS). Without sufficient sampling, it becomes challenging to assess the accuracy of values of interaction turnover, leaving uncertainty as to whether they reflect true dynamics or are merely artifacts of sampling. Therefore, the assessment of apparent interaction turnover and its components as a function of sampling effort gives us insights into whether these metrics have reached saturation and are approaching their true values, and how much sampling effort is required to reach a reliable estimate. Furthermore, analysis of seasonal trends of interaction turnover over eight years demonstrates more variable seasonal patterns from one year to the next compared to previous studies. Overall, this study contributes to a greater understanding of how sampling effort influences patterns of interaction turnover and inferences of temporal and spatial network dynamics.

## Supplementary Information

Below is the link to the electronic supplementary material.

Supplementary file1 (DOCX 4083 KB)

## Data Availability

The data supporting the results have been archived in FigShare: 10.6084/m9.figshare.25665531.

## Code availability

The code supporting the results have been archived in FigShare: 10.6084/m9.figshare.25665531.
