# Supplementary material for: Impacts of sampling effort on seasonal plant-pollinator interaction turnover over eight years
Source: Oecologia. 2025 Jul 11;207(8):131. doi: 10.1007/s00442-025-05771-8 (PMC12254060; doi:10.1007/s00442-025-05771-8)
Supplement: Supplementary file 1 — Supplementary file1 (DOCX 4083 KB) [file 442_2025_5771_MOESM1_ESM.docx]

**Supporting information for: Impacts of sampling effort on seasonal plant-pollinator interaction turnover over eight years**

Isabella Manning, Leana Zoller, & Julian Resasco


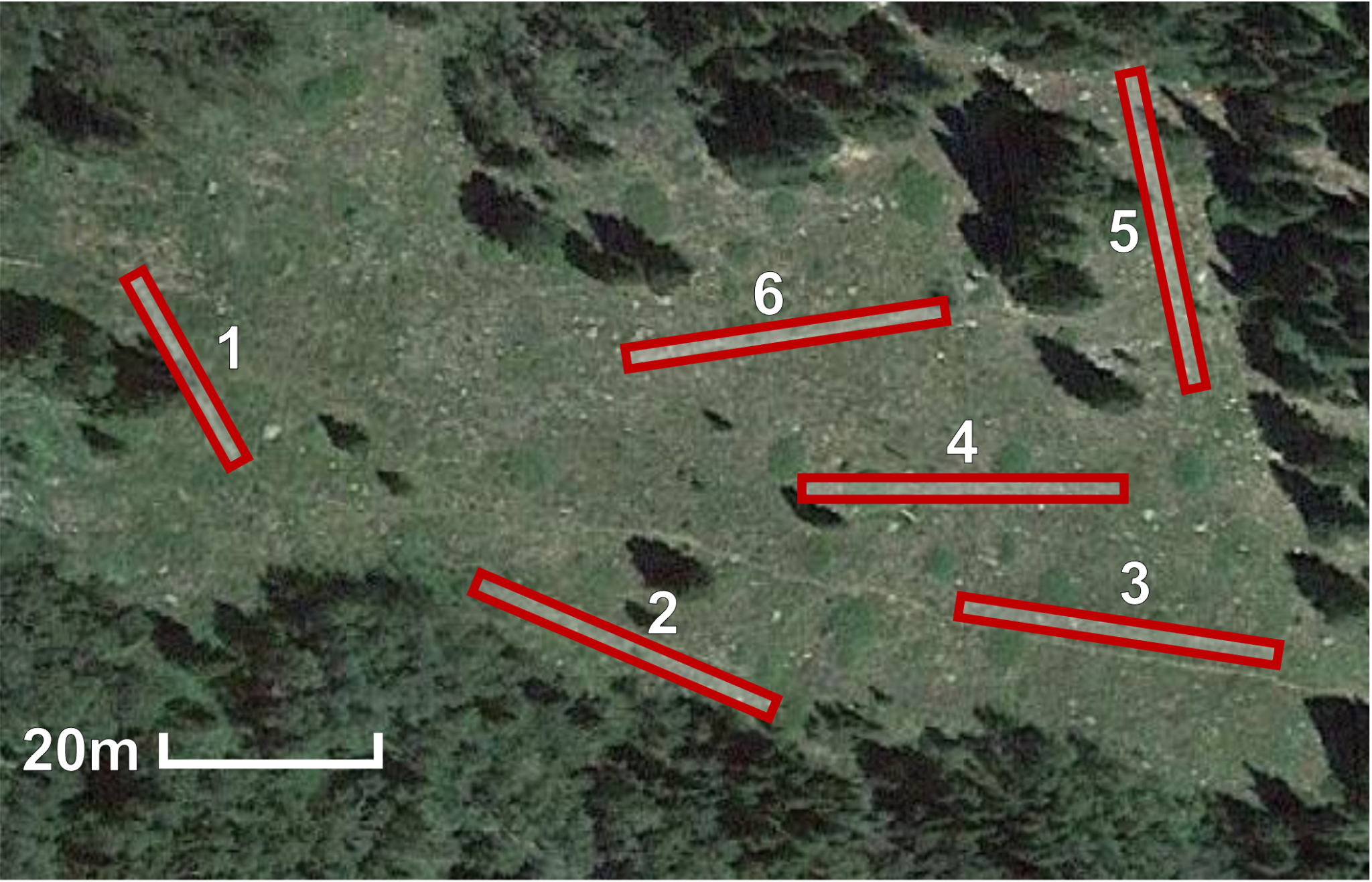


Figure S1: Six plots, 20-30 m x 2 m, Elk Meadow (meadow is approximately 110 x 40 m). Image from Google Maps.


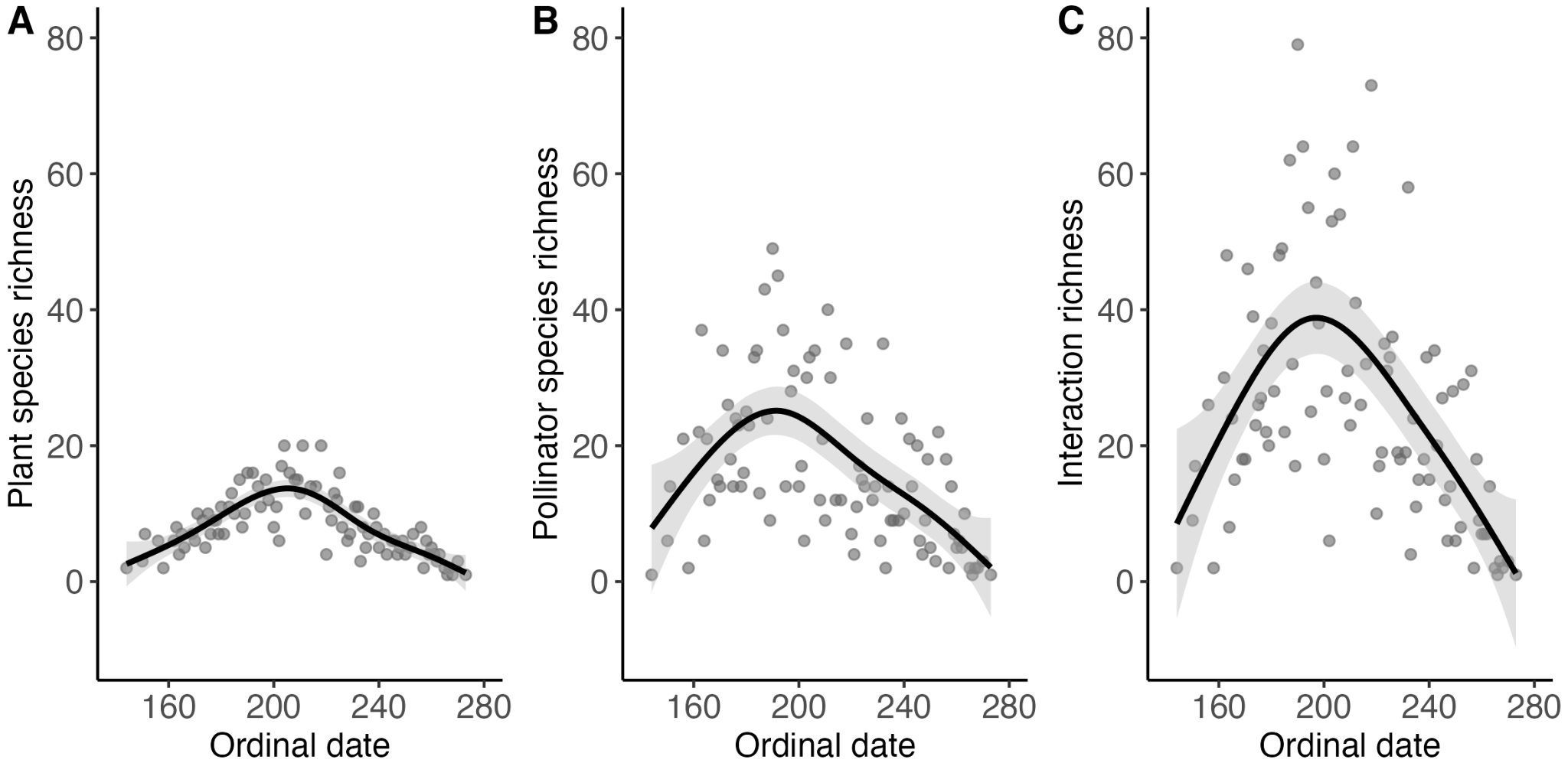
Figure S2: Seasonal trends in plant (A), pollinator (B), and interaction richness (C) across all eight years.

**
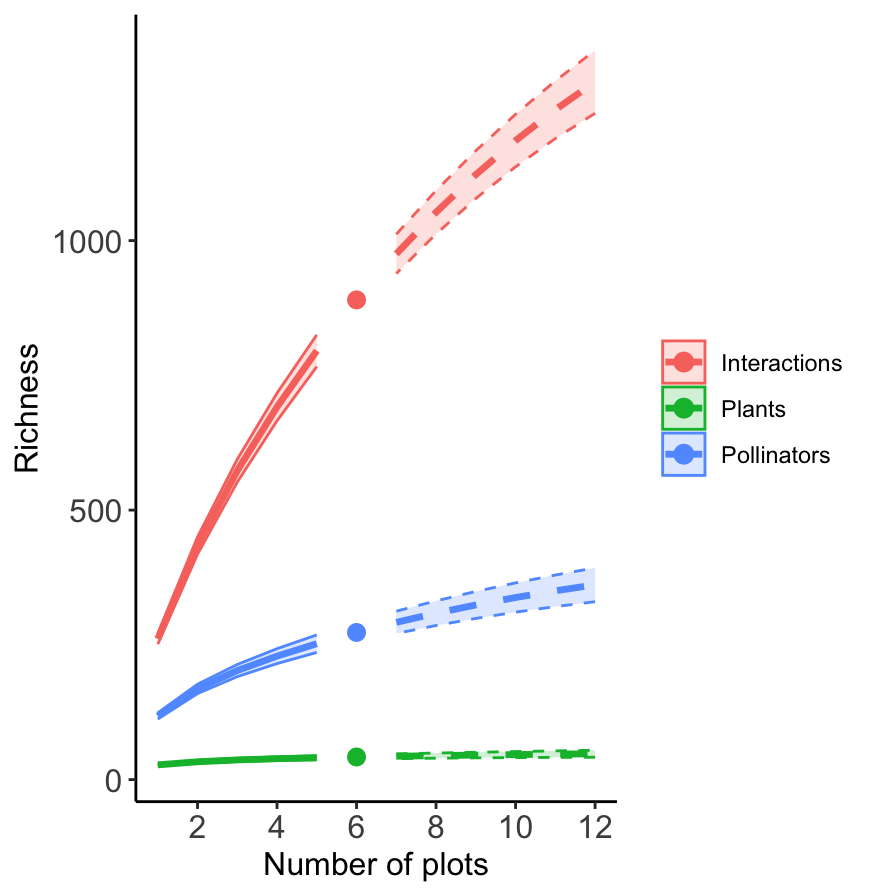
**

Figure S3: Accumulation (solid lines) and extrapolation curves (dotted lines) of plant richness, pollinator richness, and interaction richness, as sampling effort (number of plots included) increases*.*


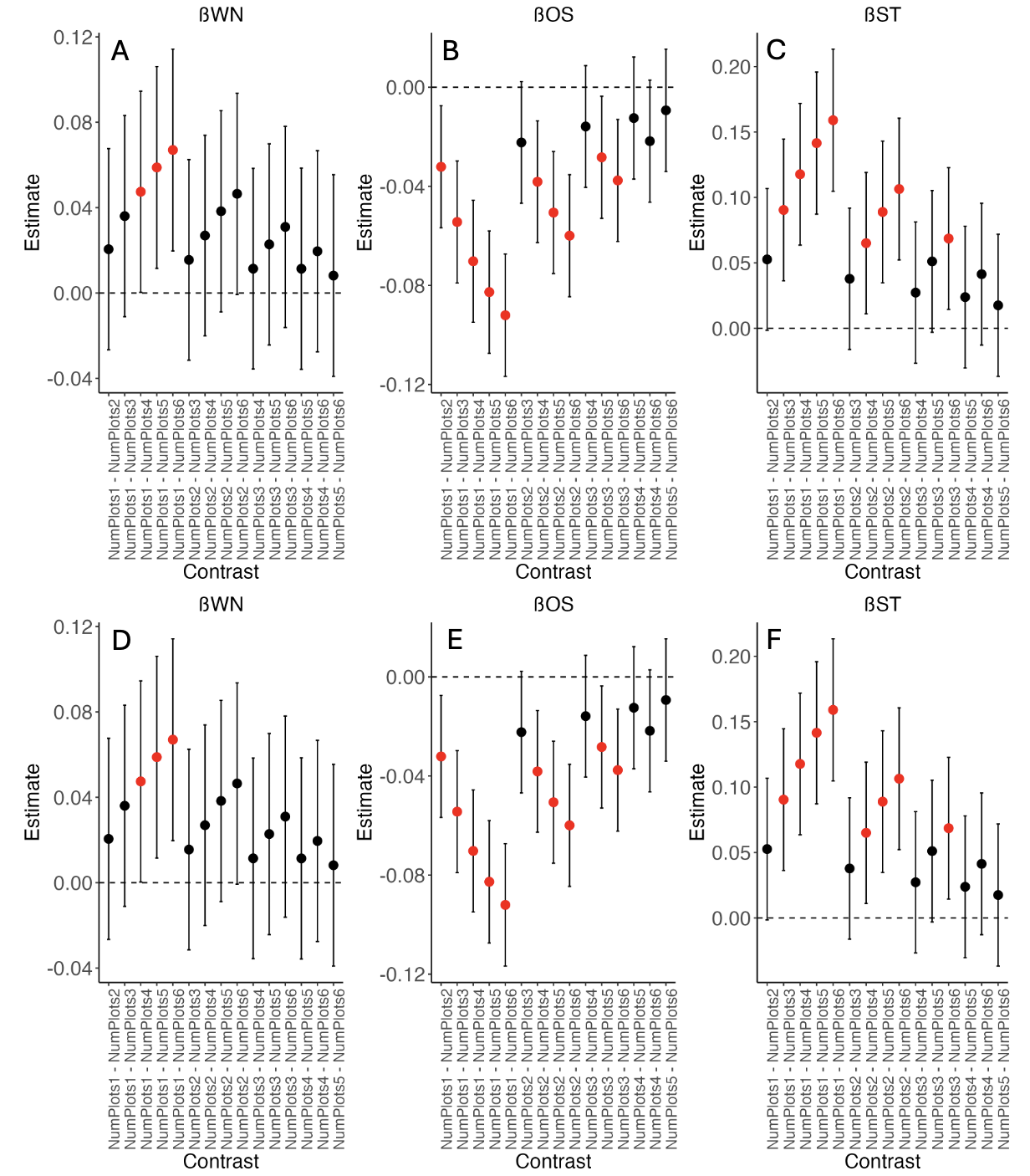
Figure S4: Comparisons of the number of plots included in the analysis and difference in estimates of interaction turnover (ßWN), interaction rewiring (ßOS), or species turnover (ßST). The top row (A, B, and C) uses the “poisot” method for partitioning. The bottom row (D, E, and F) uses the “commondenom” method for partitioning. Each pairwise comparison for the number of plots included from one through six is included on the x-axis and whether that pairwise comparison significantly affects ßWN, ßOS, and ßST is shown on the y-axis. Red dots indicate a significant relationship.

Table S1: Pairwise comparison tables using *emmeans*

| “poisot” method | | | | |
| --- | --- | --- | --- | --- |
| ***ßWN*** | | | | |

| *contrast* | *estimate* | *SE* | *df* | *lower CL* | *upper CL* | *t* | *p* |
| --- | --- | --- | --- | --- | --- | --- | --- |
| NumPlots 1 - NumPlots2 | 0.02 | 0.02 | 493 | -0.03 | 0.07 | 1.25 | 0.81 |
| NumPlots 1 - NumPlots3 | 0.04 | 0.02 | 493 | -0.01 | 0.08 | 2.19 | 0.25 |
| NumPlots 1 - NumPlots4 | 0.05 | 0.02 | 493 | 0.0003 | 0.09 | 2.88 | **<0.05** |
| NumPlots 1 - NumPlots5 | 0.06 | 0.02 | 493 | 0.01 | 0.11 | 3.56 | **<0.01** |
| NumPlots 1 - NumPlots6 | 0.07 | 0.02 | 493 | 0.02 | 0.11 | 4.06 | **<0.01** |
| NumPlots2 - NumPlots3 | 0.02 | 0.02 | 493 | -0.03 | 0.06 | 0.95 | 0.94 |
| NumPlots2 - NumPlots4 | 0.03 | 0.02 | 493 | -0.02 | 0.07 | 1.64 | 0.57 |
| NumPlots2 - NumPlots5 | 0.04 | 0.02 | 493 | -0.01 | 0.09 | 2.33 | 0.19 |
| NumPlots2 - NumPlots6 | 0.05 | 0.02 | 493 | -0.001 | 0.09 | 2.82 | 0.06 |
| NumPlots3 - NumPlots4 | 0.01 | 0.02 | 493 | -0.04 | 0.06 | 0.70 | 0.98 |
| NumPlots3 - NumPlots5 | 0.02 | 0.02 | 493 | -0.02 | 0.07 | 1.38 | 0.74 |
| NumPlots3 - NumPlots6 | 0.03 | 0.02 | 493 | -0.02 | 0.08 | 1.88 | 0.41 |
| NumPlots4 - NumPlots5 | 0.01 | 0.02 | 493 | -0.04 | 0.06 | 0.69 | 0.98 |
| NumPlots4 - NumPlots6 | 0.02 | 0.02 | 493 | -0.028 | 0.067 | 1.187 | 0.84 |
| NumPlots5 - NumPlots6 | 0.01 | 0.02 | 493 | -0.039 | 0.055 | 0.495 | 0.1 |

| “poisot” method | | | | |
| --- | --- | --- | --- | --- |
| ***ßOS*** | | | | |

| *contrast* | *estimate* | *SE* | *df* | *lower CL* | *upper CL* | *t* | *p* |
| --- | --- | --- | --- | --- | --- | --- | --- |
| NumPlots 1- NumPlots2 | -0.101 | 0.021 | 493 | -0.160 | -0.042 | -4.904 | **<0.001** |
| NumPlots1 - NumPlots3 | -0.161 | 0.021 | 493 | -0.220 | -0.102 | -7.823 | **<0.001** |
| NumPlots1 - NumPlots4 | -0.198 | 0.021 | 493 | -0.257 | -0.139 | -9.627 | **<0.001** |
| NumPlots1 - NumPlots5 | -0.220 | 0.021 | 493 | -0.279 | -0.161 | -10.657 | **<0.001** |
| NumPlots 1- NumPlots6 | -0.234 | 0.021 | 493 | -0.293 | -0.175 | -11.325 | **<0.001** |
| NumPlots2- NumPlots3 | -0.060 | 0.021 | 493 | -0.119 | -0.001 | -2.928 | **0.041** |
| NumPlots2- NumPlots4 | -0.097 | 0.021 | 493 | -0.156 | -0.039 | -4.738 | **<0.001** |
| NumPlots2- NumPlots5 | -0.119 | 0.021 | 493 | -0.178 | -0.060 | -5.784 | **<0.001** |
| NumPlots2- NumPlots6 | -0.133 | 0.021 | 493 | -0.192 | -0.074 | -6.454 | **<0.001** |
| NumPlots3 - NumPlots4 | -0.037 | 0.021 | 493 | -0.096 | 0.022 | -1.809 | 0.461 |
| NumPlots3 - NumPlots5 | -0.059 | 0.021 | 493 | -0.118 | 0.000 | -2.865 | **<0.05** |
| NumPlots3- NumPlots6 | -0.073 | 0.021 | 493 | -0.132 | -0.014 | -3.535 | **<0.01** |
| NumPlots4 - NumPlots5 | -0.022 | 0.021 | 493 | -0.081 | 0.037 | -1.061 | 0.896 |
| NumPlots4 - NumPlots6 | -0.036 | 0.021 | 493 | -0.095 | 0.023 | -1.731 | 0.512 |
| NumPlots5 - NumPlots6 | -0.014 | 0.021 | 493 | -0.073 | 0.045 | -0.668 | 0.985 |

| “poisot” method | | | | |
| --- | --- | --- | --- | --- |
| ***ßST*** | | | | |

| *contrast* | *estimate* | *SE* | *df* | *lower CL* | *upper CL* | *t* | *p* |
| --- | --- | --- | --- | --- | --- | --- | --- |
| NumPlots1 - NumPlots2 | 0.12 | 0.02 | 493 | 0.06 | 0.19 | 5.30 | **<0.001** |
| NumPlots 1 - NumPlots3 | 0.20 | 0.02 | 493 | 0.13 | 0.26 | 8.60 | **<0.001** |
| NumPlots1 - NumPlots4 | 0.25 | 0.02 | 493 | 0.18 | 0.31 | 10.72 | **<0.001** |
| NumPlots 1- NumPlots5 | 0.28 | 0.02 | 493 | 0.21 | 0.34 | 12.14 | **<0.001** |
| NumPlots1 - NumPlots6 | 0.30 | 0.02 | 493 | 0.24 | 0.37 | 13.09 | **<0.001** |
| NumPlots2- NumPlots3 | 0.08 | 0.02 | 493 | 0.01 | 0.14 | 3.31 | **0.01** |
| NumPlots2- NumPlots4 | 0.12 | 0.02 | 493 | 0.06 | 0.19 | 5.44 | **<0.001** |
| NumPlots2- NumPlots5 | 0.16 | 0.02 | 493 | 0.09 | 0.22 | 6.87 | **<0.001** |
| NumPlots2- NumPlots6 | 0.18 | 0.02 | 493 | 0.11 | 0.24 | 7.83 | **<0.001** |
| NumPlots3 - NumPlots4 | 0.05 | 0.02 | 493 | -0.02 | 0.11 | 2.13 | 0.28 |
| NumPlots3 - NumPlots5 | 0.08 | 0.02 | 493 | 0.02 | 0.15 | 3.57 | **0.01** |
| NumPlots3 - NumPlots6 | 0.10 | 0.02 | 493 | 0.04 | 0.17 | 4.53 | **<0.001** |
| NumPlots4 - NumPlots5 | 0.03 | 0.02 | 493 | -0.03 | 0.10 | 1.45 | 0.70 |
| NumPlots4- NumPlots6 | 0.06 | 0.02 | 493 | -0.01 | 0.12 | 2.41 | 0.15 |
| NumPlots5 - NumPlots6 | 0.02 | 0.02 | 493 | -0.04 | 0.09 | 0.96 | 0.93 |

| “commondenom” method | | | | |
| --- | --- | --- | --- | --- |
| ***ßWN*** | | | | |

| *contrast* | *estimate* | *SE* | *df* | *lower CL* | *upper CL* | *t* | *p* |
| --- | --- | --- | --- | --- | --- | --- | --- |
| NumPlots 1 - NumPlots2 | 0.02 | 0.02 | 493 | -0.03 | 0.07 | 1.25 | 0.81 |
| NumPlots 1 - NumPlots3 | 0.04 | 0.02 | 493 | -0.01 | 0.08 | 2.19 | 0.25 |
| NumPlots 1 - NumPlots4 | 0.05 | 0.02 | 493 | 0.0003 | 0.09 | 2.88 | **<0.05** |
| NumPlots 1 - NumPlots5 | 0.06 | 0.02 | 493 | 0.01 | 0.11 | 3.56 | **<0.01** |
| NumPlots 1 - NumPlots6 | 0.07 | 0.02 | 493 | 0.02 | 0.11 | 4.06 | **<0.01** |
| NumPlots2 - NumPlots3 | 0.02 | 0.02 | 493 | -0.03 | 0.06 | 0.95 | 0.94 |
| NumPlots2 - NumPlots4 | 0.03 | 0.02 | 493 | -0.02 | 0.07 | 1.64 | 0.57 |
| NumPlots2 - NumPlots5 | 0.04 | 0.02 | 493 | -0.01 | 0.09 | 2.33 | 0.19 |
| NumPlots2 - NumPlots6 | 0.05 | 0.02 | 493 | -0.001 | 0.09 | 2.82 | 0.06 |
| NumPlots3 - NumPlots4 | 0.01 | 0.02 | 493 | -0.04 | 0.06 | 0.70 | 0.98 |
| NumPlots3 - NumPlots5 | 0.02 | 0.02 | 493 | -0.02 | 0.07 | 1.38 | 0.74 |
| NumPlots3 - NumPlots6 | 0.03 | 0.02 | 493 | -0.02 | 0.08 | 1.88 | 0.41 |
| NumPlots4 - NumPlots5 | 0.01 | 0.02 | 493 | -0.04 | 0.06 | 0.69 | 0.98 |
| NumPlots4 - NumPlots6 | 0.02 | 0.02 | 493 | -0.028 | 0.067 | 1.187 | 0.84 |
| NumPlots5 - NumPlots6 | 0.01 | 0.02 | 493 | -0.039 | 0.055 | 0.495 | 0.1 |

| “commondenom” method | | | | |
| --- | --- | --- | --- | --- |
| ***ßOS*** | | | | |

| *contrast* | *estimate* | *SE* | *df* | *lower CL* | *upper CL* | *t* | *p* |
| --- | --- | --- | --- | --- | --- | --- | --- |
| NumPlots1 - NumPlots2 | -0.03 | 0.01 | 493 | -0.06 | -0.01 | -3.73 | **<0.001** |
| NumPlots 1 - NumPlots3 | -0.05 | 0.01 | 493 | -0.08 | -0.03 | -6.32 | **<0.001** |
| NumPlots1 - NumPlots4 | -0.07 | 0.01 | 493 | -0.09 | -0.05 | -8.16 | **<0.001** |
| NumPlots1 - NumPlots5 | -0.08 | 0.01 | 493 | -0.11 | -0.06 | -9.58 | **<0.001** |
| NumPlots 1 - NumPlots6 | -0.09 | 0.01 | 493 | -0.12 | -0.07 | -10.66 | **<0.001** |
| NumPlots2- NumPlots3 | -0.02 | 0.01 | 493 | -0.05 | 0.00 | -2.60 | 0.10 |
| NumPlots2- NumPlots4 | -0.04 | 0.01 | 493 | -0.06 | -0.01 | -4.44 | **<0.001** |
| NumPlots2- NumPlots5 | -0.05 | 0.01 | 493 | -0.08 | -0.03 | -5.88 | **<0.001** |
| NumPlots2 - NumPlots6 | -0.06 | 0.01 | 493 | -0.08 | -0.04 | -6.96 | **<0.001** |
| NumPlots3 - NumPlots4 | -0.02 | 0.01 | 493 | -0.04 | 0.01 | -1.85 | 0.44 |
| NumPlots3- NumPlots5 | -0.03 | 0.01 | 493 | -0.05 | 0.00 | -3.29 | **0.01** |
| NumPlots3 - NumPlots6 | -0.04 | 0.01 | 493 | -0.06 | -0.01 | -4.37 | **<0.001** |
| NumPlots4 - NumPlots5 | -0.01 | 0.01 | 493 | -0.04 | 0.01 | -1.45 | 0.70 |
| NumPlots4 - NumPlots6 | -0.02 | 0.01 | 493 | -0.05 | 0.00 | -2.53 | 0.12 |
| NumPlots5 - NumPlots6 | -0.01 | 0.01 | 493 | -0.03 | 0.02 | -1.08 | 0.89 |

| “commondenom” method | | | | |
| --- | --- | --- | --- | --- |
| ***ßST*** | | | | |

| *contrast* | *estimate* | *SE* | *df* | *lower CL* | *upper CL* | *t* | *p* |
| --- | --- | --- | --- | --- | --- | --- | --- |
| NumPlots 1- NumPlots2 | 0.05 | 0.02 | 493 | 0.00 | 0.11 | 2.78 | 0.06 |
| NumPlots1 - NumPlots3 | 0.09 | 0.02 | 493 | 0.04 | 0.14 | 4.78 | **<0.001** |
| NumPlots1 - NumPlots4 | 0.12 | 0.02 | 493 | 0.06 | 0.17 | 6.22 | **<0.001** |
| NumPlots 1 - NumPlots5 | 0.14 | 0.02 | 493 | 0.09 | 0.20 | 7.45 | **<0.001** |
| NumPlots 1 - NumPlots6 | 0.16 | 0.02 | 493 | 0.10 | 0.21 | 8.38 | **<0.001** |
| NumPlots2- NumPlots3 | 0.04 | 0.02 | 493 | -0.02 | 0.09 | 2.00 | 0.34 |
| NumPlots2 - NumPlots4 | 0.07 | 0.02 | 493 | 0.01 | 0.12 | 3.45 | **0.01** |
| NumPlots2- NumPlots5 | 0.09 | 0.02 | 493 | 0.03 | 0.14 | 4.70 | **<0.001** |
| NumPlots2 - NumPlots6 | 0.11 | 0.02 | 493 | 0.05 | 0.16 | 5.62 | **<0.001** |
| NumPlots3- NumPlots4 | 0.03 | 0.02 | 493 | -0.03 | 0.08 | 1.44 | 0.70 |
| NumPlots3 - NumPlots5 | 0.05 | 0.02 | 493 | 0.00 | 0.11 | 2.70 | 0.08 |
| NumPlots3 NumPlots6 | 0.07 | 0.02 | 493 | 0.01 | 0.12 | 3.62 | **<0.001** |
| NumPlots4 - NumPlots5 | 0.02 | 0.02 | 493 | -0.03 | 0.08 | 1.26 | 0.81 |
| NumPlots4 - NumPlots6 | 0.04 | 0.02 | 493 | -0.01 | 0.10 | 2.18 | 0.25 |
| NumPlots5 - NumPlots6 | 0.02 | 0.02 | 493 | -0.04 | 0.07 | 0.92 | 0.94 |

Table S2: Summary table of linear regression models for ßWN, ßOS, and ßST for each method of partitioning.

| “poisot” method | | | | |
| --- | --- | --- | --- | --- |
| ***ßWN*** | | | | |
| *Predictors* | *Estimate* | *SE* | *t* | *p* |
| (Intercept) | 0.83 | 0.01 | 70.92 | **<0.001** |
| Ordinal day | -0.28 | 0.11 | -2.64 | **0.01** |
| Ordinal day^2^ | 0.81 | 0.11 | 7.57 | **<0.001** |
| NumPlots2 | -0.02 | 0.02 | -1.25 | 0.21 |
| NumPlots3 | -0.04 | 0.02 | -2.19 | **0.03** |
| NumPlots4 | -0.05 | 0.02 | -2.88 | **<0.001** |
| NumPlots5 | -0.06 | 0.02 | -3.56 | **<0.001** |
| NumPlots6 | -0.07 | 0.02 | -4.06 | **<0.001** |
| R² / R² adjusted | 0.151 / 0.139 | | | |

| “poisot” method | | | | |
| --- | --- | --- | --- | --- |
| ***ßOS*** | | | | |
| *Predictors* | *Estimate* | *SE* | *t* | *p* |
| (Intercept) | 0.08 | 0.01 | 5.79 | **<0.001** |
| Ordinal day | -0.72 | 0.13 | -5.44 | **<0.001** |
| Ordinal day^2^ | -1.60 | 0.13 | -12.03 | **<0.001** |
| NumPlots2 | 0.10 | 0.02 | 4.90 | **<0.001** |
| NumPlots3 | 0.16 | 0.02 | 7.82 | **<0.001** |
| NumPlots4 | 0.20 | 0.02 | 9.63 | **<0.001** |
| NumPlots5 | 0.22 | 0.02 | 10.66 | **<0.001** |
| NumPlots6 | 0.23 | 0.02 | 11.32 | **<0.001** |
| R² / R² adjusted | 0.422 / 0.413 | | | |

| “poisot” method | | | | |
| --- | --- | --- | --- | --- |
| ***ßST*** | | | | |
| *Predictors* | *Estimate* | *SE* | *t* | *p* |
| (Intercept) | 0.74 | 0.02 | 45.78 | **<0.001** |
| Ordinal day | 0.44 | 0.15 | 2.98 | **0.003** |
| Ordinal day^2^ | 2.41 | 0.15 | 16.26 | **<0.001** |
| NumPlots2 | -0.12 | 0.02 | -5.30 | **<0.001** |
| NumPlots3 | -0.20 | 0.02 | -8.60 | **<0.001** |
| NumPlots4 | -0.25 | 0.02 | -10.72 | **<0.001** |
| NumPlots5 | -0.28 | 0.02 | -12.14 | **<0.001** |
| NumPlots6 | -0.30 | 0.02 | -13.09 | **<0.001** |
| R² / R² adjusted | 0.512 / 0.505 | | | |

| “commondenom” method | | | | |
| --- | --- | --- | --- | --- |
| ***ßWN*** | | | | |
| *Predictors* | *Estimate* | *SE* | *t* | *p* |
| (Intercept) | 0.83 | 0.01 | 70.92 | **<0.001** |
| Ordinal day | -0.28 | 0.11 | -2.65 | **0.01** |
| Ordinal day^2^ | 0.81 | 0.11 | 7.57 | **<0.001** |
| NumPlots2 | -0.02 | 0.02 | -1.25 | 0.21 |
| NumPlots3 | -0.04 | 0.02 | -2.19 | **0.03** |
| NumPlots4 | -0.05 | 0.02 | -2.88 | **<0.001** |
| NumPlots5 | -0.06 | 0.02 | -3.56 | **<0.001** |
| NumPlots6 | -0.07 | 0.02 | -4.06 | **<0.001** |
| R² / R² adjusted | 0.151 / 0.139 | | |  |

| “commondenom” method | | | | |
| --- | --- | --- | --- | --- |
| ***ßOS*** | | | | |
| *Predictors* | *Estimate* | *SE* | *t* | *p* |
| (Intercept) | 0.04 | 0.01 | 5.81 | **<0.001** |
| Ordinal day | -0.02 | 0.06 | -0.38 | 0.70 |
| Ordinal day^2^ | -0.59 | 0.06 | -10.64 | **<0.001** |
| NumPlots2 | 0.03 | 0.01 | 3.73 | **<0.001** |
| NumPlots3 | 0.05 | 0.01 | 6.32 | **<0.001** |
| NumPlots4 | 0.07 | 0.01 | 8.16 | **<0.001** |
| NumPlots5 | 0.08 | 0.01 | 9.58 | **<0.001** |
| NumPlots6 | 0.09 | 0.01 | 10.66 | **<0.001** |
| R² / R² adjusted | 0.357 / 0.357 | | | |

| “commondenom” method | | | | |
| --- | --- | --- | --- | --- |
| ***ßST*** | | | | |
| *Predictors* | *Estimate* | *SE* | *t* | *p* |
| (Intercept) | 0.79 | 0.01 | 59.06 | **<0.001** |
| Ordinal day | -0.26 | 0.12 | -2.13 | **0.03** |
| Ordinal day^2^ | 1.40 | 0.12 | 11.43 | **<0.001** |
| NumPlots2 | -0.05 | 0.02 | -2.78 | **0.01** |
| NumPlots3 | -0.09 | 0.02 | -4.78 | **<0.001** |
| NumPlots4 | -0.12 | 0.02 | -6.22 | **<0.001** |
| NumPlots5 | -0.14 | 0.02 | -7.45 | **<0.001** |
| NumPlots6 | -0.16 | 0.02 | -8.38 | **<0.001** |
| R² / R² adjusted | 0.321 / 0.312 | | | |

Table S3: Summary table of linear mixed effect models for ßWN, ßOS, and ßST for both partitioning methods.

| “poisot” method | | | | | |
| --- | --- | --- | --- | --- | --- |
| ***ßWN*** | | | | | |
| *Predictors* | *Estimate* | *SE* | *df* | *t* | *p* |
| (Intercept) | 0.75 | 0.02 | 6.87 | 38.23 | **<0.001** |
| Ordinal day | -0.30 | 0.10 | 99.89 | -2.95 | **<0.001** |
| Ordinal day^2^ | 0.00 | 0.10 | 99.63 | -0.03 | 0.98 |

| “poisot” method | | | | | |
| --- | --- | --- | --- | --- | --- |
| ***ßOS*** | | | | | |
| *Predictors* | *Estimate* | *SE* | *df* | *t* | *p* |
| (Intercept) | 0.36 | 0.03 | 6.61 | 14.07 | **<0.001** |
| Ordinal day | -0.13 | 0.17 | 100.26 | -0.77 | 0.45 |
| Ordinal day^2^ | -0.32 | 0.17 | 99.87 | -1.91 | 0.058 |

| “poisot” method | | | | | |
| --- | --- | --- | --- | --- | --- |
| ***ßST*** | | | | | |
| *Predictors* | *Estimate* | *SE* | *df* | *t* | *p* |
| (Intercept) | 0.39 | 0.02 | 6.75 | 17.58 | **<0.001** |
| Ordinal day | -0.17 | 0.15 | 100.42 | -1.18 | 0.24 |
| Ordinal day^2^ | 0.31 | 0.15 | 100.04 | 2.16 | **0.03** |

| “commondenom” method | | | | | |
| --- | --- | --- | --- | --- | --- |
| ***ßWN*** | | | | | |
| *Predictors* | *Estimate* | *SE* | *df* | *t* | *p* |
| (Intercept) | 0.75 | 0.02 | 6.87 | 38.23 | **<0.001** |
| Ordinal day | -0.30 | 0.10 | 99.89 | -2.95 | **<0.001** |
| Ordinal day^2^ | 0.00 | 0.10 | 99.63 | -0.03 | 0.98 |

| “commondenom” method | | | | | |
| --- | --- | --- | --- | --- | --- |
| ***ßOS*** | | | | | |
| *Predictors* | *Estimate* | *SE* | *df* | *t* | *p* |
| (Intercept) | 0.14 | 0.01 | 6.41 | 17.89 | **<0.001** |
| Ordinal day | 0.14 | 0.08 | 101.75 | 1.80 | 0.08 |
| Ordinal day^2^ | -0.15 | 0.08 | 101.21 | -1.98 | 0.051 |
|  |  |  |  |  |  |
| “commondenom” method | | | | | |
| ***ßST*** | | | | | |
| *Predictors* | *Estimate* | *SE* | *df* | *t* | *p* |
| (Intercept) | 0.60 | 0.02 | 6.92 | 28.72 | **<0.001** |
| Ordinal day | -0.44 | 0.12 | 100.24 | -3.57 | **<0.001** |
| Ordinal day^2^ | 0.14 | 0.12 | 99.92 | 1.17 | 0.24 |
